# Socioeconomic Status and Overweight/obesity in an Adult Chinese Population in Singapore

**DOI:** 10.2188/jea.17.161

**Published:** 2007-09-01

**Authors:** Charumathi Sabanayagam, Anoop Shankar, Tien Yin Wong, Seang Mei Saw, Paul J. Foster

**Affiliations:** 1 Department of Community, Occupational, and Family Medicine, Yong Loo Lin School of Medicine, National University of Singapore.; 2 Centre for Eye Research Australia, University of Melbourne.; 3 Singapore National Eye Centre & Singapore Eye Research Institute.; 4 Department of Epidemiology, Institute of Ophthalmology, University College London.

**Keywords:** Body Mass Index, Overweight, Obesity, Prevalence, Socioeconomic Factors, Chinese, Singapore

## Abstract

**BACKGROUND:**

Studies from industrialized Western countries have reported an inverse association between socioeconomic status and overweight/obesity. In contrast, few studies from newly industrialized countries in Asia have examined this association. In this context, we examined the association between socioeconomic status and overweight/obesity by gender in Chinese adults in Singapore.

**METHODS:**

A population-based cross sectional study of 942 participants (57.3% women, 40-81 years) residing in the Tanjong Pagar district of Singapore was conducted. Education, income, and housing type were used as socioeconomic status indicators. Main outcome-of-interest was the presence of overweight/obesity (n=313), classified by body mass index as overweight (25-29.9 kg/m^2^), or obese (≥30 kg/m^2^)

**RESULTS:**

The prevalence of overweight/obesity was 33% in men and 34% in women. In men, SES indicators were not associated with overweight/obesity. In women, SES indicators were found to be inversely associated with overweight/ obesity. Compared to women with secondary/higher education, the odds ratio (OR) (95% confidence interval [CI]) of overweight/obesity in women with primary/lower education was 2.5 (1.5-4.0). Compared to women earning > Singapore dollar (SGD) 1,000 per month, the OR (95% CI) of overweight/obesity among women earning ≤SGD 1,000 was 2.5 (1.4-4.5). Compared to women living in large size public apartments or private houses, the OR (95% CI) of overweight/obesity in women living in small/medium size public apartments was 1.8 (1.2-2.7).

**CONCLUSIONS:**

Lower socioeconomic status, defined by education, income, and housing type was associated with overweight/obesity in Chinese Singaporean women.

Obesity is a global epidemic with the prevalence of obesity increasing rapidly in both developed and developing countries.^1^ In recent years there has been considerable interest in examining socioeconomic variations in the prevalence of overweight/ obesity.^2^^-^^9^ Studies have shown that the association between socioeconomic status (SES) and overweight/obesity may vary by economic development of populations, by gender, and by age.^2^^,^^7^^,^^10^^-^^13^ The pattern of association varies by economic development of a country. In industrialized countries, persons of lower SES groups are more likely to be overweight/obese than their higher SES counterparts,^2^^,^^4^^,^^10^ whereas in developing countries, high SES groups are more likely to be overweight/obese.^1^^,^^13^^-^^15^ However, few previous studies have examined the relationship between SES and overweight/obesity in newly industrialized societies from Asia such as Singapore, Taiwan, Hong Kong, and South Korea, where the prevalence of overweight/obesity is increasing.^16^^-^^19^ Also, some previous studies have suggested that the health impact of SES may not be the same in men and women.^20^^-^^23^ In affluent Western societies, studies have reported an inverse relationship between SES factors and obesity among women; similar relationship was not evident among men.^2^^,^^7^^,^^12^^,^^21^^,^^24^^,^^25^ In contrast, in developing societies, a strong and direct relationship exists between SES factors and overweight/obesity both among men and women.^2^^,^^15^^,^^26^ Studies examining the relationship between SES and overweight/obesity focusing on gender differences from industrialized Asian countries are limited.

Singapore is a newly industrialized Asian country with approximately 76% of the population comprising of individuals of Chinese ethnicity.^27^ A significant proportion of older Singaporean residents are first generation migrants from southern China. In the present study we examined the association between overweight/obesity and SES measured by educational level, income and housing type, by gender, in a population-based sample of Singapore adults.

## METHODS

### Study Population

The Tanjong Pagar survey was a population-based cross-sectional survey of adult Chinese aged 40-81 years residing in the Tanjong Pagar district in Singapore. The survey was conducted between October 1997 and August 1998. Detailed population selection and methodology have been previously reported.^28^^-^^30^ In brief, the 1996 Singapore electoral register in the district of Tanjong Pagar was used as the sampling frame in this study. The electoral register listed 15,082 Chinese names aged between 40 and 79 years residing in the district. Two thousand (13.3%) names were selected using a disproportionate (with more weights given to the older age groups), stratified, clustered, random sampling method. This involved selecting residents randomly, 500 from each of 4 age strata: 40-49, 50-59, 60-69 and 70-79 years, residing in 50 area clusters defined by street name (out of a total of 84), located within specified boundaries of the pre-designated study clinic. The area clusters selected were those with the largest concentration of persons in the district (82% of the population). Among the 2,000 names selected, 46 had died and 235 had moved to addresses outside the district before the study period, and 2 people were excluded on grounds of ill health, leaving 1,717 subjects considered eligible to participate in this study. Among the 1717 subjects eligible for the survey, 1232 (71.8% of eligible subjects) participated, and 1,090 (63.4%) attended the clinic examination. The number of persons with data on body mass index (BMI) was 1,082. The final sample included for analysis, after eliminating persons with missing information on the SES variables considered, was 942. Compared to those who were excluded in the final analysis, those who were included were younger, taller, heavier, had higher education levels, more likely to be professionals and office workers, production operators or salespeople, lived in better housing, and had higher individual income.

### Measures

Height was measured in centimeters (cm) and converted to meters for calculation of BMI and weight in kilograms (kg) on a single automatic weighing scale with the person standing up without shoes. BMI was derived from the ratio of person's weight divided by the square of his height and recorded in kg/m^2^. Questionnaires were used to collect information on participant's age, self-reported individual monthly income, highest year of schooling completed, housing type, and current smoking. Alcohol consumption was rare in this population, and therefore not studied.

### Definitions

BMI was used to classify adults as overweight (25- 29.9 kg/m^2^), or obese (≥30 kg/m^2^).^31^ Age was defined as the age at the time of examination. Participants' age was categorized into 4 groups: 40-49, 50-59, 60-69, and 70-81 years. A standard index of socioeconomic status has not been developed in Singapore. We included educational attainment, individual monthly income and housing type as indicators of SES. Although occupation was also assessed, it was not included as a SES indicator in our study, because it was not available for all individuals, and occupation data collected on homemakers and retirees were imprecise. Furthermore, the occupational status as classified in Singapore is not ranked for socioeconomic status. Also, compared to occupation, educational attainment has previously been shown to be a better SES indicator in examining health related outcomes^32^ and assessing cardiovascular risk factors.^33^ Persons were classified by educational level into three categories: (1) primary or lower (≤6 years), (2) secondary (7-10 years), and (3) post-secondary (≥11 years, including university education). Individual monthly income was based in Singapore dollars ((SGD) (approximate exchange rate of SGD1.5 = USD1.0)) and was ascertained by the question, "What is your monthly income (before tax is subtracted)?" with the response recorded into one of nine groups, but recategorized into three groups for analysis: (1) low (≤SGD1000), (2) middle (SGD1001-2000), and (3) high (>SGD2000). Housing type was classified as follows: (1) small size public apartments (1-2 room), (2) medium size public apartments (3 room), and (3) large size public apartments (4-5 room) or private housing. Smoking status was categorized into current smoking and non-smoking.

### Statistical Analysis

We performed separate analyses for men and women. Descriptive analyses were performed for all variables and differences between men and women using analysis of variance or chi square test, as appropriate. Because the prevalence of obesity in the study population was low, we combined overweight and obesity categories to obtain adequate sample size. Unadjusted prevalence of overweight/obesity with 95% confidence intervals (CIs, using CIPROP programme) by each SES indicator was calculated. The association of education, income, and housing type with overweight/obesity was assessed using logistic regression models after controlling for significant confounding factors (age and smoking status); variables also considered as potential confounding factors, but not included in the final model included diabetes (absent, present), hypertension (absent, present) and systolic and diastolic blood pressures (mm Hg). Tests for trend were performed using each SES indicator as ordinal variable. Odds ratios (ORs) and 95% CIs of overweight/obesity were estimated by using the highest SES category as the reference for each SES indicator. Statistical interaction between gender and each SES indicator was examined in the corresponding logistic regression model by including cross-product interaction terms. All reported p values were based on two sided tests and compared to a significance level of 5%. Statistical interaction was deemed significant if p-interaction was <0.10. All analyses were performed using SAS^®^ version 9.1.

### Ethical Issues

Informed written consent was obtained from all participants, and ethics approval was obtained from the Singapore National Eye Centre.

## RESULTS

Demographic and behavioral characteristics of the study population and the distribution of SES indicators by gender are shown in Table 1. The study involved predominantly older individuals (average age=58.0 years). Men were significantly more likely to be current smokers, to have higher educational achievement, and a higher income. Spearman correlation for men and women between income and education was 0.51 and 0.57, between income and housing type was 0.39 and 0.24, and between education and housing was 0.39 and 0.32.

**Table 1.** Characteristics of participants in the Tanjong Pagar Study, Singapore.*

Characteristic	Both genders (n=942)	Men (n=402)	Women (n=540)	p-value*
Age (year)	58.0 (11.0)	57.6 (11.0)	58.3 (11.1)	0.33
Weight (kg)	59.2 (11.7)	64.1 (12.1)	55.5 (9.8)	<0.01
Height (cm)	158.1 (8.4)	164.9 (6.3)	153 (5.7)	<0.01
Body mass index (kg/m^2^)^†^	23.6 (3.8)	23.5 (3.7)	23.7 (3.9)	0.48
Overweight/obesity (%)^‡^	33.2 (30.2-36.3)	32.6 (28.0-37.4)	33.7 (29.7-37.9)	0.72
Current Smoker (%)	19.1 (16.6-21.8)	35.3 (30.6-40.2)	7.0 (5.0-9.5)	<0.01
Educational level (%)				
Primary or lower	65.4 (62.3-68.4)	57.2 (52.2-62.1)	71.5 (67.5-75.3)	<0.01
Secondary	26.8 (23.9-29.7)	32.8 (28.3-37.6)	22.2 (18.8-26.0)	
Post-secondary	7.9 (6.2-9.8)	10.0 (7.2-13.3)	6.3 (4.3-8.7)	
Income (%)^§^				
Low (≤1000 SGD)	68.2 (65.1-71.1)	49.2 (44.3-54.3)	82.2 (78.7-85.3)	<0.01
Middle (1001-2000 SGD)	19.6 (17.1-22.3)	32.6 (28.0-37.4)	10.0 (7.6-12.8)	
High (>2000 SGD)	12.2 (10.2-14.5)	18.2 (14.5-22.3)	7.8 (5.7-10.3)	
Housing (%)^|^				
Small	18.4 (15.9-21.0)	20.4 (16.6-24.7)	16.9 (13.8-20.3)	0.37
Medium	53.7 (50.5-56.9)	52.7 (47.7-57.7)	54.4 (50.1-58.7)	
Large/private	27.9 (25.1-30.9)	26.8 (22.6-31.5)	28.7 (24.9-32.7)	

* : P-value for the difference in characteristics by gender based on chi-square test or analysis of variance, as appropriate. Data presented are means (standard deviations [SD]) or proportions (95% confidence interval), as appropriate for the variable.

† : Body Mass Index (BMI) was calculated as weight (in kg) divided by height (in m^2^).

‡ : Overweight/obesity was defined as BMI ≥ 25 Kg/m^2^.

§ : Income was based on individual monthly income in Singapore dollars (SGD).

| : Housing: (1) small size public apartments (1-2 room), (2) medium size public apartments (3 room), and (3) large public apartments (4-5 room) or private housing.

In the whole cohort, 33% were overweight/obese, with a mean BMI of 23.6kg/m^2^ (standard deviation of 3.8kg/m^2^). The mean BMI decreased with increasing levels of education, (p-trend <0.0001). Current smokers had lower BMI. Increasing age categories had a lower prevalence of overweight/obesity and lower BMI, (p-trend <0.05). In a linear regression analysis, BMI was found to be inversely related to increasing age categories (indicator variable), after adjusted for education (β = −0.041, SE=0.012, p=0.0007)

The prevalence of overweight/obesity by SES indicators and by gender is shown in Table 2. An inverse relationship was observed between the level of education and the prevalence of overweight/obesity in women (p<0.01). Prevalence of overweight/obesity was highest (38%) among the primary or lower educated women and lowest (12%) among women with post-secondary education (Table 2). In men, the prevalence of overweight/obesity was also lowest (23%) among those with post-secondary education. Among women, income was significantly associated with overweight/obesity (p<0.05). The prevalence of overweight/obesity was highest (37%) among low income earners among women. Among the 3 housing types we examined, majority (54%) of the study population lived in medium housing (3 room public apartments) in both genders. In women the lowest prevalence of overweight/obesity (25%) was observed among those with large housing (>3 room or private housing). These associations with education, income, and housing type were not observed in men.

**Table 2.** Prevalence of overweight/obesity by socioeconomic status (SES) and other selected factors in men and women.

	Men (n=402)			Women (n=540)		
	
	No. at risk	Mean BMI* (SD)	Prevalence (%) of overweight (95% CI)*	No. at risk	Mean BMI* (SD)	Prevalence (%) of overweight (95% CI)*
Educational level						
Primary or lower	230	23.4(3.9)	33 (27 - 40)	230	23.4(3.9)	33 (27 - 40)
Secondary	132	24.0(3.3)	35 (27 - 44)	132	24.0(3.3)	35 (27 - 44)
Post-secondary	40	22.5(3.4)	23 (11 - 38)	40	22.5(3.4)	23 (11 - 38)

Income(SGD)^†^						
Low (≤1000)	198	23.0(3.9)	28 (22 - 35)	198	23.0(3.9)	28 (22 - 35)
Middle (1001-2000)	131	23.9(3.6)	38 (30 - 47)	131	23.9(3.6)	38 (30 - 47)
High (>2000)	73	24.1(3.2)	36 (25 - 48)	73	24.1(3.2)	36 (25 - 48)

Housing^‡^						
Small	82	22.3(4.1)	23 (15 - 34)	82	22.3(4.1)	23 (15 - 34)
Medium	212	23.7(3.6)	35 (29 - 42)	212	23.7(3.6)	35 (29 - 42)
Large/Private	108	23.9(3.5)	34 (25 - 44)	108	23.9(3.5)	34 (25 - 44)

Age (year)						
40-49	119	23.9(3.4)	34 (25 - 43)	119	23.9(3.4)	34 (25 - 43)
50-59	99	24.2(3.7)	38 (29 - 49)	99	24.2(3.7)	38 (29 - 49)
60-69	115	23.5(3.9)	36 (27 - 45)	115	23.5(3.9)	36 (27 - 45)
70-81	69	21.7(3.5)	17 (9 - 28)	69	21.7(3.5)	17 (9 - 28)

Current Smoking						
No^§^	260	24.0(3.5)	38 (32 - 44)	260	24.0(3.5)	38 (32 - 44)
Yes	142	22.6(3.9)	23 (17 - 31)	142	22.6(3.9)	23 (17 - 31)

Total sample	402	23.5(3.7)	33 (28 - 37)	402	23.5(3.7)	33 (28 - 37)

* : BMI: Body Mass Index (kg/m2); CI: confidence interval

† : Income was based on individual monthly income in Singapore dollars (SGD).

‡ : Housing: (1) small size public apartments (1-2 room), (2) medium size public apartments (3 room), and (3) large public apartments (4-5 room) or private housing.

§ : Non-smoking and previous smoking

Table 3 shows the OR of overweight/obesity in relation to SES by gender. Among women, in separate analyses, lower categories of educational level, income, and housing were positively associated with overweight/obesity; similar association was not observed in men. To formally evaluate the observed effect modification of SES and overweight/obesity by gender, we included cross product interaction terms in the corresponding multivariable models. There was a significant gender × educational status interaction (p-interaction=0.03), gender × income interaction (p-interaction=0.003), and gender × housing type interaction (p-interaction=0.03).

**Table 3.** Odds ratios (ORs) and their 95% confidence intervals (Cis) for the prevalence of overweight/obesity in relation to socioeconomic status (SES) in men and women.

	Men (n=402)							
	
	No. at risk	No. of cases	Unadjusted OR (95% CI)	Adjusted OR* (95% CI)	No. at risk	No. of cases	Unadjusted OR (95% CI)	Adjusted OR* (95% CI)
SES category								
Educational level^§^								
Primary or lower	230	76	1.1 (0.7-1.6)	1.4 (0.9-2.3)	386	147	2.1 (1.4-3.2)	2.5 (1.5-40)
Secondary or higher	172	55	1.0 (Reference)	1.0 (Reference)	154	35	1.0 (Reference)	1.0 (Reference)
p value			0.82	0.13			<0.01	<0.01

Income(SGD)^†§^								
Low (≤1000)	198	55	0.7 (0.4-1.0)	0.8 (0.5-1.3)	444	162	2.2 (1.3-3.7)	2.5 (1.4-45)
Middle and High (>1000)	204	76	1.0 (Reference)	1.0 (Reference)	96	20	1.0 (Reference)	1.0 (Reference)
p value			0.04	0.41			<0.01	<0.01

Housing^‡§^								
Small and Medium	294	94	0.9 (0.6-1.4)	1.0 (0.6-1.7)	385	143	1.8 (1.2-2.7)	1.8 (1.2-2.7)
Large/Private	108	37	1.0 (Reference)	1.0 (Reference)	155	39	1.0 (Reference)	1.0 (Reference)
p value			0.66	0.91			<0.01	<0.01

* : Adjusted for age and smoking status

† : Income was based on individual monthly income in Singapore dollars (SGD)

‡ : Housing: small size public apartments (1/2 room), medium size public apartments (3 room), and large size public apartments (> 3 room) or private housing

§ : P-interaction for sex and income=0.003, sex and education=0.03, and sex and housing=0.03

We performed several sets of supplementary analyses. First, we examined the association between SES variables and overweight/ obesity after excluding men and women in the oldest age group (70-81 years). Among women, the multivariable OR (95% CI) of overweight/ obesity was 2.33 (1.43-3.79) for educational level (primary or lower vs. secondary or higher [referent]), 2.32 (1.31-4.13) for income (≤SGD1000 vs. >SGD1000 [referent]), and 1.94 (1.22-3.08) for housing (small and medium size public housing type vs. large size public apartments or private houses [referent]). Among men, the multivariable OR (95% CI) of overweight/ obesity was 1.40 (0.86-2.29) for educational level, 0.91 (0.54-1.52) for income, and 1.04 (0.62-1.72) for housing.

In a second supplementary analysis, we tested the association of employment with overweight/obesity. In women the prevalence of overweight/obesity was 37.7% among unemployed and 30.4% among employed participants (p=0.11) whereas in men the prevalence was 31.6% among unemployed and 32.6% among employed participants (p=0.92). The multivariable OR (95% CI) of overweight/obesity for employment (unemployed vs. employed [referent]) was 1.43 (0.98-2.09) among women and 0.95 (0.35-2.62) among men. In another supplementary analysis, we performed stratified analysis by age category (<60 years, ≥60 years); the overall results were similar to Table 3. Finally, in a supplementary analysis that incorporated sampling weights in the logistic regression models, the results were similar.

## DISCUSSION

In this study, data from a representative sample of Chinese population aged 40-81 years in Singapore suggests that low SES, defined by categories of education, income, and housing, was associated with a higher prevalence of overweight/obesity in women. In men, no significant relations between these SES indicators and overweight/obesity were found.

The overall prevalence of overweight/obesity in the current study was 33%, similar to previous reports from national surveys in Singapore conducted by the Ministry of Health (33% in 2004 and 30% in 1998).^16^^,^^34^ In the present study, current smoking was inversely associated with overweight/obesity, a finding similar to previous studies.^35^^-^^37^ These consistent findings with previous studies indirectly suggest that our results have reasonable internal validity.

In the current study, SES was found to be inversely associated with overweight/obesity among adult Singaporean women. In our study, we used commonly used measures of SES such as education, income, and housing to assess the prevalence of overweight/obesity. These findings from Singapore, a newly industrialized Asian country, are similar to the previously reported inverse association between SES variables and overweight/obesity in developed Western societies.^2^^,^^7^^,^^21^^,^^24^^,^^25^ Also, as expected, these results are dissimilar to reports of a direct, positive association between SES and overweight/obesity in developing countries from Asia.^15^^,^^26^

Previous studies in adults show that SES is one of the most consistent correlates of body weight.^2^^,^^7^^,^^38^^-^^40^ SES factors such as education, income, and closely related occupation are related to variations in behaviors which change energy consumption, energy expenditure and metabolism.^41^ Education enables people to integrate healthy behaviors (e.g., specific dietary patterns, lack of exercise), into a coherent lifestyle, gives them a sense of control over their health^42^ and limits exposure to negative influences associated with the social and physical environment in which one lives and works.^43^ Income may reflect access to medical care resources, good housing and working conditions, and provides opportunities for healthy lifestyles.^44^ Also higher SES is positively associated with weight control behaviors such as physical activity, access to healthy foods, and less time spent watching television.^2^^,^^45^^-^^47^ Based on our findings, a corollary observation is that the previously reported association between low SES and mortality^48^ may be explained, at least in part, by the association between low SES and overweight/ obesity.

In the current study, we found that an inverse association between SES factors and overweight/obesity was present only in women, but not in men. These are consistent with previous studies on gender differences in the risk of overweight/obesity and SES classes.^20^^-^^22^^,^^24^^,^^39^^,^^49^ In a prospective birth cohort study, adult social classes were inversely related to overweight/obesity among women, but not among men.^24^ In the US Third National Health and Nutrition Examination Survey (NHANES III), 1988-1994, a stronger, inverse association between SES and obesity was observed in women compared with men.^21^ The KORA survey 2000 conducted in Germany, found a stronger inverse association between BMI and SES indicators including education, income, and occupational status in women. In men, these associations were weaker or absent.^49^ In the 1998 Korean National Health and Nutrition Examination Survey, education was inversely associated with obesity in women, but not in men.^22^

The observed gender differences in the relation between SES and overweight/obesity may have a number of plausible explanations. In developed/industrialized societies, men and women may have different attitudes towards body weight and have different practices for controlling body weight.^45^^,^^50^^,^^51^ In most Western societies, women hold a more negative attitude towards overweight/obesity than men.^50^ Social pressure for slimness is stronger among women than men, particularly among high SES women,^2^ with associated dieting behavior^45^ and with more physical activity.^52^ Furthermore, the absence of association between low SES and overweight/obesity in men could be explained by the fact that low SES men were more likely to have physically demanding occupations, which reduced the risk of obesity, than high SES men. Also, the association of smoking with a lower BMI^35^^-^^37^ may contribute to inconsistent associations between socioeconomic position and BMI among men^2^ particularly given our crude measure of smoking.

Our findings are also similar to studies involving adolescents, which showed a strong inverse relationship of SES to overweight prevalence in white adolescent females.^12^^,^^53^ Similar analogous findings are noted in cardiovascular studies where low SES exerts a stronger adverse influence on cardiovascular risk factors of women than it does on those of men^54^^,^^55^ and in diabetic studies where a negative association between SES and prevalence of diabetes was found only among women.^49^^,^^56^

Not all studies have consistently shown these gender patterns. In the 1996 Health Survey in England, higher educational attainment was associated with a lower risk of obesity/overweight in both men and women, although higher occupational status was associated with a lower risk only among women.^20^ An inverse association between obesity and education was found only in men in Finland.^57^

Because of the cross-sectional nature of the survey, it is difficult to determine whether there is a temporal relationship between SES and overweight/obesity. We do not have information on physical activity or other relevant lifestyle factors which could confound the observed association. Also the lack of information on household income, marital status, or the size of the family would be a potential limitation, because SES of women would more likely to be influenced by these factors. Further, it is possible that the associations seen in our Chinese population in Singapore may differ from other ethnic groups, with dissimilar genetic and environmental exposures, different distributions of height and weight and higher rates of obesity. The strengths of our study includes the use of population based sampling strategy, the use of measured rather than self-reported heights and weights to calculate BMI, the availability of several indicators of SES, and the inclusion of potentially confounding variables in the multivariate analysis. We believe that our study sample is fairly representative of the adult Chinese population in Singapore for the following reasons: (1) Tanjong Pagar district is centrally located in Singapore and includes a representative range of social and economic backgrounds and housing types, and (2) we selected subjects using a variation of the stratified, random sampling strategy from the population-based electoral register which is regularly updated by Elections department under the Prime Minister's office and almost 100% complete.^58^

In conclusion, lower SES as defined by education, income and housing type was associated with a greater risk of overweight/obesity only in women. In men no significant associations between SES indicators and overweight/obesity were found. The observation of gender differences and SES differences in overweight/obesity warrants further research to investigate the underlying reasons and to plan appropriate public health intervention strategies.
