# RE: “Socioeconomic status and overweight/obesity in an adult Chinese population in Singapore”

**DOI:** 10.2188/jea.18.43

**Published:** 2008-02-28

**Authors:** C Sabanayagam, A Shankar, TY Wong, SM Saw, PJ Foster

About the paper by Sabanayagam C et al. published in the September 2007 issue of the *Journal*,^1^ the authors requested that some errors in the Tables 2 (p164) and 3 (p165) of the original paper should be corrected. The revised version of the Tables are following.

The *Journal* regrets the errors.

**Table 2.** Prevalence of overweight/obesity by socioeconomic status (SES) and other selected factors in men and women.

	Men (n=402)			Women (n=540)		

	No. at risk	Mean BMI* (SD)	Prevalence (%) of overweight (95% CI)*	No. at risk	Mean BMI* (SD)	Prevalence (%) of overweight (95% CI)*
Educational level						
Primary or lower	230	23.4(3.9)	33 (27 - 40)	386	24.0(4.0)	38 (33 - 43)
Secondary	132	24.0(3.3)	35 (27 - 44)	120	23.2(3.5)	26 (18 - 35)
Post-secondary	40	22.5(3.4)	23 (11 - 38)	34	21.7(3.7)	12 ( 3 - 27)

Income(SGD) ^†^						
Low (≤1000)	198	23.0(3.9)	28 (22 - 35)	444	23.9(3.9)	37 (32 - 41)
Middle (1001-2000)	131	23.9(3.6)	38 (30 - 47)	54	23.0(3.5)	19 ( 9 - 31)
> High ( 2000)	73	24.1(3.2)	36 (25 - 48)	42	22.4(3.6)	24 (12 - 39)

Housing ^‡^						
Small	82	22.3(4.1)	23 (15 - 34)	91	23.4(4.0)	32 (23 - 43)
Medium	212	23.7(3.6)	35 (29 - 42)	294	24.0(4.0)	39 (33 - 45)
Large/Private	108	23.9(3.5)	34 (25 - 44)	155	23.1(3.7)	25 (19 - 33)

Age (year)						
40-49	119	23.9(3.4)	34 (25 - 43)	136	23.2(4.4)	28 (21 - 36)
50-59	99	24.2(3.7)	38 (29 - 49)	167	24.1(3.4)	40 (32 - 47)
60-69	115	23.5(3.9)	36 (27 - 45)	126	24.1(4.0)	37 (29 - 46)
70-81	69	21.7(3.5)	17 ( 9 - 28)	111	23.0(3.7)	28 (20 - 37)

Current Smoking						
No ^§^	260	24.0(3.5)	38 (32 - 44)	502	23.7(3.8)	34 (30 - 38)
Yes	142	22.6(3.9)	23 (17 - 31)	38	22.7(4.8)	32 (18 - 49)

Total sample	402	23.5(3.7)	33 (28 - 37)	540	23.7(3.9)	34 (30 - 38)

* : BMI: Body Mass Index (kg/m^2^); CI: confidence interval

^†^ : Income was based on individual monthly income in Singapore dollars (SGD).

^‡^: Housing: (1) small size public apartments (1-2 room), (2) medium size public apartments (3 room), and (3) large public apartments (4-5 room) or private housing.

§ : Non-smoking and previous smoking

**Table 3.** Odds ratios (ORs) and their 95% confidence intervals (Cis) for the prevalence of overweight/obesity in relation to socioeconomic status (SES) in men and women.

SES category	Men (n=402)				Women (n=540)			

	No. at risk	No. of cases	Unadjusted OR (95% CI)	Adjusted OR* (95% CI)	No. at risk	No. of cases	Unadjusted OR (95% CI)	Adjusted OR* (95% CI)
Educational level ^§^								
Primary or lower	230	76	1.1 (0.7-1.6)	1.4(0.9-2.3)	386	147	2.1(1.4-3.2)	2.5 (1.5-4.0)
Secondary or higher	172	55	1.0 (Reference)	1.0 (Reference)	154	35	1.0 (Reference)	1.0 (Reference)
p value			0.82	0.13			< 0.01	< 0.01

Income(SGD) ^†§^								
Low ( ≤1000)	198	55	0.7 (0.4-1.0)	0.8 (0.5-1.3)	444	162	2.2 (1.3-3.7)	2.5 (1.4-4.5)
Middle and High (>1000)	204	76	1.0 (Reference)	1.0 (Reference)	96	20	1.0 (Reference)	1.0 (Reference)
p value			0.04	0.41			< 0.01	< 0.01

Housing ^‡§^								
Small and Medium	294	94	0.9 (0.6-1.4)	1.0 (0.6-1.7)	385	143	1.8 (1.2-2.7)	1.8 (1.2-2.7)
Large/Private	108	37	1.0 (Reference)	1.0 (Reference)	155	39	1.0 (Reference)	1.0 (Reference)
p value			0.66	0.91			< 0.01	< 0.01

* : Adjusted for age and smoking status

† : Income was based on individual monthly income in Singapore dollars (SGD)

‡ : Housing: small size public apartments (1/2 room), medium size public apartments (3 room), and large size public apartments (> 3 room) or private housing

§ : P-interaction for sex and income=0.003, sex and education=0.03, and sex and housing=0.03
